# Rate of herbicide resistant weed development: A Canadian Prairie case study

**DOI:** 10.1080/21645698.2025.2477231

**Published:** 2025-03-09

**Authors:** Chelsea Sutherland, Savannah Gleim, Simona Lubieniechi, Stuart J. Smyth

**Affiliations:** Department of Agricultural Economics, University of Saskatchewan, Saskatoon, Canada

**Keywords:** GM crops, herbicides, sustainability, tillage, weed control

## Abstract

Genetically modified crop adoption in Canada has been the key driver in removing tillage as the lead form of weed control, due to increased weed control efficiency. Land use has transitioned from the use of summerfallow to continuous cropping, predominantly involving zero or minimum tillage practices. Prairie crop rotations have diversified away from mainly cereals to include three-year rotations of cereals, pulses, and oilseeds. Total herbicide volume applied has increased as crop production acres increased, but the rate of herbicide active ingredient applied per hectare has declined. Diverse crop rotations allow for weed control using herbicides with different modes of action, reducing selection pressure for resistant weed development. Herbicide-resistant weeds are an important concern for farmers, as the loss of key herbicides would make weed control exceedingly more difficult. The objective of this case study is to examine herbicide resistance weed development in the Canadian Prairies and to identify changes in resistance development following GM crop adoption.

## Introduction

Herbicide resistance (HR) in weeds in Canada dates back to the 1950s and has been an issue of leading importance for farmers, as increased weed management efficiency is an integral part of Canadian agriculture’s sustainability improvement. Herbicide-resistant weeds are a global problem, not one that is specific to the Canadian Prairies. The development of HR in weed populations increased as the use of herbicides became more common in crop production through the latter half of the twentieth century. In part, this development was driven by the dryland, monoculture crop production on the Canadian Prairies that was commonly one crop followed by summerfallow, such as wheat-summerfallow.^1^ In this form of land management, the consistent use of identical herbicides would be common.

**Figure 1.**
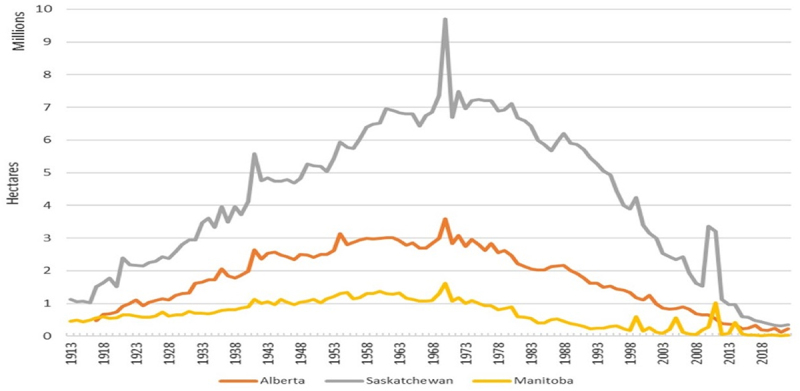
Summerfallow area in the Canadian Prairies, 1913–2022.

**Source**: Statistics Canada, 2022.^2^

The commercialization of genetically modified (GM) herbicide-tolerant (HT) crops revolutionized farmers’ approaches to weed management in the 1990s. With the adoption of GMHT crops, especially canola, in-crop weed control became so effective that Prairie farmers continually transitioned summerfallow and the accompanying tillage out of their land management practices. Transitioning summerfallow out of crop rotations is common in many GM crop-adopting countries and is especially the case in Canada. Figure 1 illustrates that summerfallow acres have dramatically declined and now represent less than 5% of crop production acres across the three Prairie Provinces.^2^ With the decline in summerfallow practices, the Canadian Prairies also witnessed increased crop types grown in rotations, with many rotations now a variation of cereal–pulse–oilseed,^3^ which also precipitated a change in herbicide use. The total volume of herbicide use increased, as farmers moved toward zero tillage systems and decreased summerfallow and, at the same time, the total amount of active ingredient applied per hectare as well as their environmental impacts has decreased over the past 25 years.^4^

Resistance in weed populations develops after repeated use of the same herbicide active ingredient or mode of action (MOA), which refers to the mechanism in the plant that the herbicide negatively impacts or inhibits.^5^ Herbicide resistance mechanisms in weeds involve either target site (TS) or non-target site (NTS) resistance, or a combination of both.^6^ Target site resistance mechanisms involve alteration of a gene that interferes with the target protein or enzyme’s ability to absorb the applied herbicide, while NTS resistance does not involve the target protein and instead refers to mechanisms that reduce the overall level of herbicide that reaches the target site.^7,8^ Because NTS resistance is not specific to a herbicide’s target site and can therefore impact efficacy of herbicides in multiple groups, these resistance mechanisms are more difficult to manage and study.^9^

Some herbicide formulations contain multiple active ingredients or modes of action (MOA) to control multiple weed species and/or mitigate resistance. To combat the development of resistant weeds, farmers have been encouraged to employ effective, diverse, and integrated weed management strategies using herbicides and non-herbicidal methods. Physical prevention of weed seed dispersal, diversifying crop rotations, shifting agronomic practices such as seeding dates or row spacings, and mechanical weed control through tillage are just some non-herbicidal methods farmers might integrate into their weed control strategies in addition to herbicide use. The challenge of using tillage to control weeds is that it contributes to increased soil erosion, allowing residues to more easily enter watersheds. Rotating herbicide MOA and using tank mixtures of multiple herbicides are strategies that help to limit the development of HR weeds. Furthermore, effective weed control strategies that minimize weed populations, whether resistant or not, not only decrease populations in which resistance can develop but also minimize the potential for weeds to go to seed and multiply.^5^

The issue of HR weeds has become a significant challenge for farmers in the United States (US).^10^ Between 1990 and 2015, the average occurrence of new resistant weed cases was approximately five per year.^11^ More specifically, the occurrence of glyphosate-resistant weeds has become a challenge for many US farmers.^12^ Adoption of GMHT crops rapidly increased upon their introduction, with GMHT corn varieties reaching 50% of acreage after ten years and adoption of GMHT cotton and GMHT soybean surpassing 50% of acreage six years post-commercialization. Over 90% of current US corn, upland cotton, and soybean acreage is planted to GM varieties that have HT, insect resistance, or both traits.^13^

Correspondingly, the reliance on glyphosate as the main form of weed control rapidly increased, especially in cotton and soybean crops, as did the occurrence of glyphosate-resistant weeds in the first decade of HT crop production.^14^ From 2005 to 2015, herbicide diversity began to increase once again in both soybean and cotton, likely in response to the development of glyphosate-resistant weeds and the need for better weed control.^11^ Yet, despite the issue of glyphosate resistance in weeds being of concern for farmers, Kniss^11^ points out that the increasing reliance on glyphosate, which has a relatively low risk of resistance development, has replaced the use of other herbicides more likely to cause resistance issues. As farmers have moved from relying on glyphosate as a burn-off prior to seeding in two-year rotations of wheat-summerfallow, to using glyphosate as an in-crop herbicide in GMHT crops, the risk remains low in instances where cereal–pulse–oilseed rotations are followed.

On the Canadian Prairies, numerous HR weed surveys have been conducted which monitor the development of resistant weeds (e.g.,^15–18^). These studies illustrate an increase in the frequency of HR weed occurrences, specifically in kochia and wild oat populations. For example, in the 2001–2003 Prairie HR weed survey, 11% of fields surveyed for HR wild oat populations contained HR biotypes and 53% of fields where viable seed was collected contained HR kochia.^19^ By 2007–2009, 44% of fields sampled for HR wild oat populations contained HR biotypes^15^ and Group 2 resistant kochia was found in 85% of surveyed fields.^20^

While the history, development, and frequency of HR weeds, especially of the most commonly reported HR weed species such as wild oat, kochia, and green foxtail, are well documented for many geographical areas, including much of the US, less research exists on the rate of development of all new HR weed species, specifically in Western Canada. The objective of this case study is to analyze the development of new HR weeds on the Canadian Prairies over the past 35 years and to compare these trends with corresponding changes in crop rotation and land management practices to assess what impact, if any, farm management practices may have had on HR development. The analysis also examines the relationship between the commercialization of GMHT crops and HR development to assess the impact this technology has had on resistant weed populations.

## Methodology

Data for this analysis were collected from two sources. Weed resistance data for the three Prairie Provinces were collected from the Herbicide Resistance Action Committee’s International Herbicide-Resistant Weed Database.^12^ The database documents cases of herbicide-resistant weeds, including new cases in specific countries or provinces. The database likely underreports new resistance cases to some degree, as not all cases may be reported; however, it is important to note that no dataset is ever fully representative of natural circumstances. Furthermore, this dataset only considers new cases of resistance, including MOA or combinations of MOAs not previously recorded, but does not document the frequency or distribution of these resistant biotypes. This vast dataset provides the opportunity to outline the history and current state of the issue of HR weeds on the Prairies and to examine the resistant species and MOA most commonly reported as problematic. Herbicide-resistant weed data were cleaned and sorted by province, weed species, and MOA before analysis. The descriptive analysis was completed using Excel software.

Prairie crop rotation and herbicide use data were collected through the University of Saskatchewan Crop Rotation Survey, an online survey of Prairie crop farmers, from 2020 to 2021. In the survey, farmers reported their land management practices on a single field, if possible, from the 1991–1994 period and/or the 2016–2019 period depending on the years they were actively farming. Participants took two to five hours, on average, to complete the survey and received up to $200 in compensation upon successful survey completion. Questions in the survey were divided into four sections, including seeding and harvest, tillage, fertilizer, and chemical applications. The survey responses used for this analysis focused on the crops and varieties planted, as well as farmers’ summerfallow practices and herbicide use.

Crop and rotational management data from the Crop Rotation Survey were sorted by year and province. After cleaning the survey data to remove incomplete or inconsistent responses, there were 94 responses to the 1991–1994 crop and rotational management questions. Eighty percent of respondents were from Saskatchewan, with the remaining 14% and 6% from Manitoba and Alberta, respectively. There were 186 total responses to the 2016–2019 survey questions, with 81% from Saskatchewan, 11% from Manitoba, and 8% from Alberta. The majority of respondents being from Saskatchewan is due, in part, to the survey being initially launched only in Saskatchewan before being opened up to farmers in all three of the Prairie Provinces the following year.

Herbicide use data from the Crop Rotation Survey were sorted by herbicide timing, active ingredients applied, and year. Due to low response volumes from Manitoba and Alberta, only responses from Saskatchewan farmers were included in this section. Variations in the timings of participants’ herbicide applications between the years under study results in inconsistent response numbers for each application timing. For 1991–1994, responses for pre-seed, in-crop, and post-harvest herbicide applications ranged from 7–68 depending on the year and the application timing. For application timings in 2016–2019, the number of responses ranged from 36–94.

## Results and Discussion

### Development of Herbicide-Resistant Weeds on the Canadian Prairies

The first HR weeds on the Canadian Prairies were reported in 1988, with four reports of new resistant weeds occurring across the three Prairie Provinces. New incidents are counted as any weed species showing a resistance mechanism, resistance to a MOA or combination of multiple MOAs, which has not been previously recorded in that specific province. Between 1988 and 2021, 66 new resistance incidents were reported in 21 different weed species on the Prairies. The 66 incidents reported over the past 35 years were spread evenly across the Prairie Provinces, with 24 in Alberta, 20 in Saskatchewan, and 22 in Manitoba. Fourteen of the 66 resistant incidences occurred in wild oat populations (*Avena fatua*), nine in kochia (*Kochia scoparia*), and nine in green foxtail (*Setaria viridis*) (Table 1). Four weed species, wild oat, kochia, green foxtail, and false cleavers (*Galium spurium*), have developed resistance to multiple MOAs. Wild oat has developed resistance to five MOAs, the most among any weed species, followed by kochia with resistance to four different MOAs and green foxtail with resistance to three.

**Table 1.** Number of new HR mechanisms on the Canadian Prairies by weed species, 1988–2021.

Scientific Name	Common Name	Resistance Cases	Cases of Multiple MOA Resistance
*Avena fatua*	Wild Oat	14	6
*Kochia scoparia*	Kochia	9	5
*Setaria viridis*	Green Foxtail	9	2
*Sinapis arvensis*	Wild Mustard	5	0
*Thlaspi arvense*	Field Pennycress	3	0
*Stellaria media*	Common Chickweed	3	0
*Galium spurium*	False Cleavers	3	1
*Galeopsis tetrahit*	Common Hempnettle	3	0
*Salsola tragus*	Russian-thistle	2	0
*Polygonum lapathifolium*	Pale Smartweed	2	0
*Lolium persicum*	Persian Darnel	2	0
*Capsella bursa-pastoris*	Shepherd’s-purse	2	0
*Amaranthus retroflexus*	Redroot Pigweed	2	0
*Vaccaria hispanica*	Cowcockle	1	0
*Sonchus asper*	Spiny Sowthistle	1	0
*Polygonum convolvulus (=Fallopia convolvulus)*	Wild Buckwheat	1	0
*Neslia paniculata*	Ball Mustard	1	0
*Chenopodium album*	Common Lambsquarters	1	0
*Bromus tectorum*	Downy Brome	1	0
*Amaranthus powellii*	Powell Amaranth	1	0
**Total**		**66**	**14**

**Figure 2.**
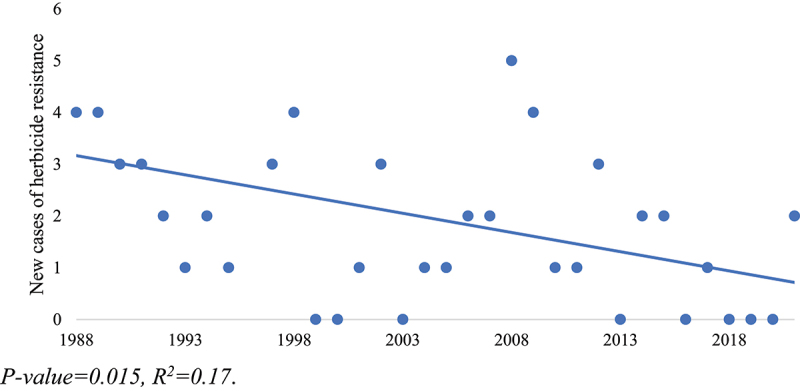
Number of reports of new HR weed incidences on the Prairies per year, 1988–2021.

**Figure 3.**
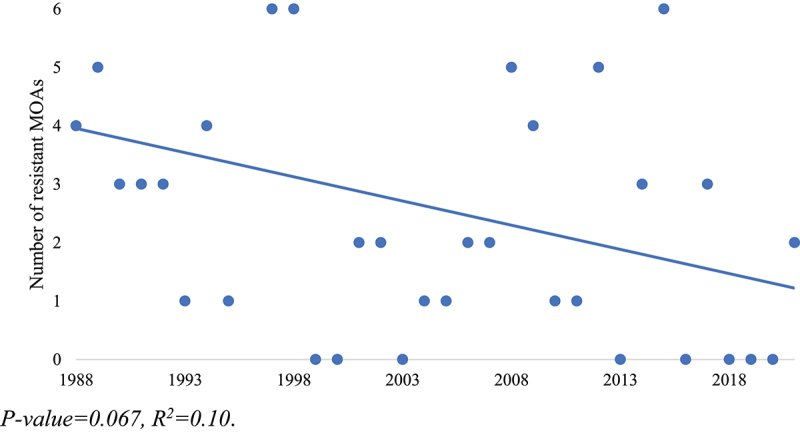
Number of resistant MOAs among weed species in Prairie HR cases per year, 1988–2021, including cases of multiple resistant MOAs within one species.

Despite the fear that the introduction and rapid adoption of GM crops would exacerbate the problem of HR weeds, the number of new resistance cases has stayed relatively stable and exhibits a slight downward trend (*p* < .05) over the past 35 years (Figure 2). Simply examining the number of incidents, however, does not take into account the number of herbicide MOA that weeds have formed resistance to, as some weed populations have developed resistance to multiple MOAs. When the number of resistant MOAs within each resistant weed population is counted, the total MOA involved in the new resistance cases across the Prairies between 1988 and 2021 is 88. This number includes 14 instances of multiple MOAs ranging from two to four resistant MOAs per weed. The number of new resistant MOAs also trends slightly downward over the past 35 years (Figure 3), although this trend is not significant at the 95% confidence level (*p* = .067) with an R^2^ value of 0.10.

*P*-value = .015, R^2^ = 0.17.

P-value = .067, R^2^ = 0.10.

**Figure 4.**
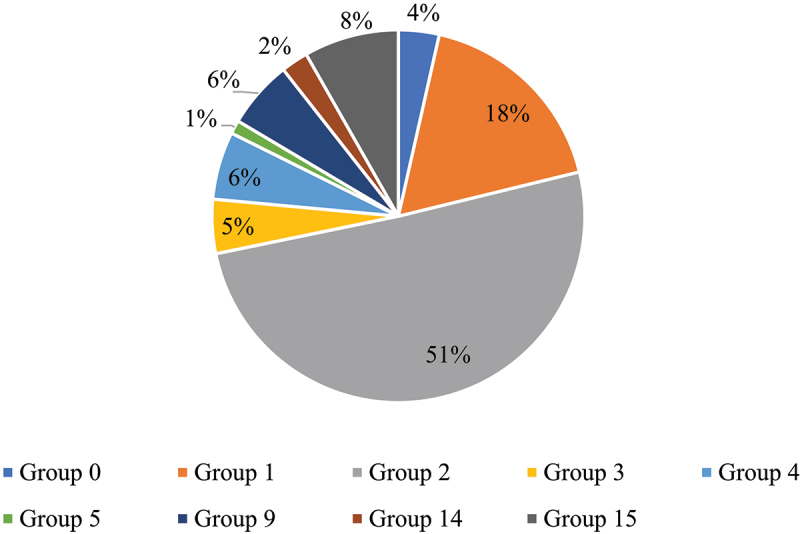
Percent of MOAs reported in HR weed species on the Prairies by herbicide group.

Between 1988 and 2021, weeds have developed resistance to ten different herbicide groups on the Prairies. However, 68% of the resistant MOAs reported in the total new herbicide resistance cases belong to two herbicide groups in particular, Group 1 and Group 2 (Figure 4), while the remaining 32% belong to one of the remaining groups. In Beckie et al.,^19^ Group 1 and 2 herbicides are classified as high risk, meaning herbicide resistance could potentially develop after ten or fewer applications, as opposed to 11–20 applications for moderate-risk herbicide groups and more than 20 applications for low-risk herbicide groups. The specific inhibition targets of the MOAs included in Figure 4 are listed in Table 2.^12^

**Table 2.** Inhibition targets of herbicide groups.

Herbicide Group	Inhibition Target
Group 0	Cell Elongation Inhibitors
Group 1	Inhibition of Acetyl CoA Carboxylase
Group 2	Inhibition of Acetolactate Synthase
Group 3	Inhibition of Microtubule Assembly
Group 4	Auxin Mimics
Group 5	PSII Inhibitors - Serine 264 Binders
Group 9	Inhibition of Enolpyruvyl Shikimate Phosphate Synthase
Group 14	Inhibition of Protoporphyrinogen Oxidase
Group 15	Very Long-Chain Fatty Acid Synthesis inhibitors

Source: Heap, 2024.^12^

Group 1 herbicides are fatty acid inhibitors first introduced in the late 1970s. The introduction of this herbicide group helped to transition applications from almost entirely pre-emergent to include in-crop applications, leading to their rapid uptake and subsequent discovery of resistance issues in wild oat populations.^21^ Group 2 herbicides, introduced in the 1980s, are amino acid inhibitors. The combination of their widespread adoption, specific mode of action, and ability to persist in the soil makes these herbicides prone to resistance challenges as well, especially in kochia and wild oats.^21^ While the widespread adoption and nature of these herbicides contribute to the higher reports of Group 1 and 2 resistance in weeds, it could also be that resistance to these herbicides is tested for most frequently, potentially biasing our results toward these resistant weeds.

When evaluating changes in resistant organisms, fitness costs must be considered. Fitness costs related to resistance development refer to costs of adaptation or deleterious effects of resistant mutations.^22^ It is rare to find HR plants within herbicide unselected weed populations, suggesting that mutations which lead to HR may result in fitness tradeoffs that limit the plant’s competitive ability under natural selection.^22^ For example, a resistant mutation that occurs within a target enzyme of a weed species may also interfere with normal functionality of the plant, resulting in a cost of adaptation that makes the resistant plant less competitive in the absence of herbicide selection.^23^ However, the expression and magnitude of these costs are impacted by many factors such as plant species, mutation type, fitness cost dominance, and environmental conditions.^24^ Furthermore, fitness costs can be lessened in successive generations through adaptation or further genomic changes.^23,24^ Fitness costs may factor into the slower than anticipated expansion of new resistant weed populations illustrated by the present analysis, as in the absence of herbicide applications for which resistance has developed, the resistant plants may not be competitive in some instances.

### Herbicide Resistance and the Introduction of GMHT Canola

**Figure 5.**
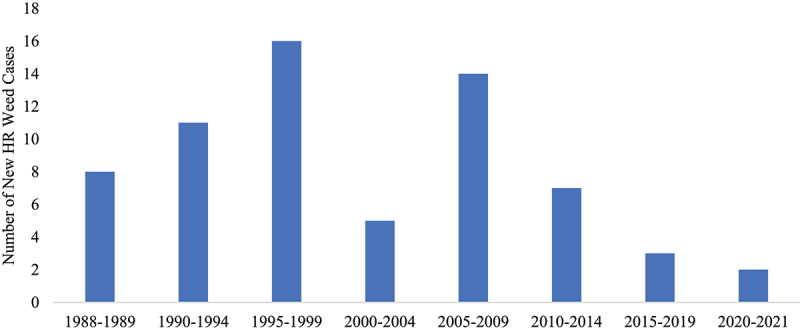
Incidence of newly reported HR weeds per 5-year period, 1988–2021.

When the reported incidents of HR weeds are examined in relation to the introduction and adoption of GMHT canola,^a^ the results help to illustrate how this technological change impacted the rate of HR development in weeds (Figure 5). It is important to note that HR data were only first available in 1988, limiting the scope of the pre-GMHT analysis to only seven years. In the years prior to the introduction of GMHT canola, there were 19 reports of new resistant weeds on the Prairies. The number of new incidents increased in the years from 1995 to 1999 as the technology was adopted and farmers transitioned summerfallow and tillage out of their land management practices. The number of new HR weeds dropped to only five between 2000 and 2004. In 2004, there were still 3.47 million hectares of summerfallow on the Prairies, which declined to 590,000 in 2019. By 2004, adoption rates of HT canola reached 98% in Western Canada.^25^ In the five years following the near-full adoption of HT canola, the number of reported HR weeds increased slightly before falling to only five between 2015 and 2021.

Of the 66 reported cases of new resistance mechanisms in weeds in the Prairies between 1988 and 2021, only five (8%) include resistance to Group 9, the herbicide group containing glyphosate. Comparatively, there are 183 occurrences of resistance to Group 9 herbicides in the US across all crop types.^12^ When GMHT crops were first introduced, many feared that the repeated use of the same chemicals, especially glyphosate, would result in “super weeds,” a term with no technical definition but often used in the media and by critics of modern farming to refer to herbicide-resistant weeds, particularly concerning the use of GM crops.^11^ Only two weed species, kochia and downy brome, have developed resistance to glyphosate on the Prairies since 1988, compared to 17 species that have developed glyphosate resistance in the US.^11^ There are currently no weed species in Canada with reported resistance to Group 10 herbicides, the group used in glufosinate-resistant canola cropping systems.^12^

Although the sole reliance on glyphosate has certainly resulted in resistance development in many cases, especially in the US where glyphosate-resistant weeds pose a significant challenge,^26^ the results indicate that the nearly complete adoption of GMHT canola on the Prairies has not led to significant HR weed issues to date. One of the reasons for this is that there are three different HT canola technologies^b^ in the market that are tolerant to glyphosate, glufosinate, and imidazolinone. Farmers rotate HT canola varieties such that they are applying one of these three herbicides, rather than consistently applying the same herbicide. Furthermore, the effective in-crop weed control provided by HT cropping systems allows farmers to move away from frequent use of Group 1 and Group 2 herbicides, for which resistance development is at a much higher risk.^27^

Recently, canola varieties containing tolerance to both glyphosate and glufosinate have been commercialized.^c^ Production of these varieties allows farmers to apply two different modes of action to the same canola field to achieve effective weed control,^28^ further diversifying farmers’ weed control strategies within GMHT canola systems. Concerns of difficulty controlling volunteer populations surfaced with the commercialization of these stacked trait canola varieties. However, with the appropriate use of tank mixes, reported challenges with volunteer populations have not increased in response to the commercialization of these varieties as of yet.^29^ Responsible use of these stacked trait varieties, including the use of crop and herbicide rotation, as well as continued use of appropriate tank mixing strategies, will help to limit potential for challenges controlling volunteer plants in the future.

### Changes in Prairie Farmers’ Crop Rotation and Management Practices

There are many possible reasons why the number of new HR weed cases has not exploded in the way that environmental activist organizations predicted following the introduction and adoption of HT crops. It may be that the weeds with the highest potential to develop resistance, such as kochia, wild oat, and green foxtail, had already developed resistance to the commonly used herbicide MOAs and there are simply fewer opportunities for resistance to develop. It could be, in part, because glyphosate and glufosinate, the two herbicides that are safe to spray in-crop on GMHT canola varieties, pose a lesser risk for HR development than the herbicides they are replacing.^27^ Yet, the adaptability and improved weed management strategies of Prairie farmers are important factors to consider. Examining how crop rotation and weed management practices have changed over the past 35 years in parallel with the adoption of HT crops illustrates how farmers have done their part in combatting the development of HR weeds.

There are various ways farmers can diversify their weed management practices, but longer and more diverse crop rotations are one of the most effective methods. The expansion and diversification of crop rotations allow farmers to also rotate the pesticides they apply to their fields. Depending on the crop kind that is planted, expansion of crop rotation does not necessitate diversification of herbicide use.^30^ Rotating between crops that require the same active ingredients applied for weed control, such as glyphosate-resistant canola and glyphosate-resistant soybean, will continue to provide selection pressure for the same weeds. However, crop rotations practiced with the intention of diversifying weed control strategies and active ingredients used are effective methods for minimizing the risk of HR weed development.

Results from the 2020 Crop Rotation Survey indicate that, between 1991–1994, only 30% of Prairie farmers (*n* = 94) included a pulse crop, either peas or lentils, in their rotations compared to 58% by 2016–2019 (*n* = 186). Beyond the inclusion of pulse crops, general diversification of crop types planted helps to expand not only crop rotations but the accompanying herbicide rotations as well, as each crop type has different herbicide options best suited to its production. Between 1991–1994 and 2016–2019, the percentage of farmers that included three or more crop types in their four-year rotations increased from 59% to 80%. Over the same period, the average number of crop types planted in the four-year rotations increased from 2.6 to 3.1. The practice of summerfallow also decreased from inclusion in 39% to only 2% of four-year rotations between 1991–1994 and 2016–2019.

Rotation of herbicides is especially important in HT crops where continuous use of one herbicide can increase selection pressure for weeds resistant to that MOA. For HT canola crops, this can include alternating between varieties tolerant to glyphosate, glufosinate, or imidazolinone. Of farmers in the survey who planted canola more than once in their rotation between 2016 and 2019 (*n* = 57), 39% reported rotating between HT genetics, while 53% used the same HT trait and an additional 9% chose not to specify the varieties they planted.

Responses from the chemical use section of the Crop Rotation Survey indicate that over the past 30 years, Saskatchewan farmers have simultaneously diversified their crop rotations as well as the MOA used in their herbicide applications. For pre-seed, in-crop, and post-harvest herbicide applications, the average number of farmers applying multiple active ingredients per application has increased by 37%, 8%, and 22%, respectively, between 1991–1994 and 2016–2019. Table 3 shows how the number of different MOAs used by the sample of survey respondents has expanded and diversified between the time periods. While glyphosate (Group 9) is still predominantly used across all timings in both periods, the increase in other herbicide groups, especially for pre-seed and post-harvest applications, indicates that Saskatchewan farmers are expanding their herbicide selections to include other active ingredients, an important component of sustainable weed management strategies.

**Table 3.** Change in number of herbicide MOAs applied to field crops in Saskatchewan, 1991–1994 and 2016–2019.

1991–1994			2016–2019		
Pre-Seed	In-Crop	Post-Harvest	Pre-Seed	In-Crop	Post-Harvest
Group 3	Group 1	Group 3	Group 2	Group 1	Group 2
Group 4	Group 2	Group 4	Group 3	Group 2	Group 3
Group 9	Group 3	Group 9	Group 4	Group 4	Group 4
	Group 4		Group 6	Group 5	Group 8
	Group 5		Group 9	Group 6	Group 9
	Group 6		Group 14	Group 7	Group 14
	Group 8		Group 15	Group 9	Group 19
	Group 9			Group 10	
	Group 10			Group 22	
				Group 27	

### Crop Rotation and Management Practices in the United States

In comparison, crop rotations in the US are generally less complex than in the Canadian Prairies. One 2024 study of rotational complexity and yield found that the most commonly practiced rotation in the major corn, cotton, soybean, and winter wheat producing areas of the US is a two-year alternation of corn-soybean. When combining this rotation type with variations including two consecutive years of either corn or soybean before alternating, these crop rotations compose more than 40% of all US cropland. Furthermore, approximately 9% of US cropland is managed in the continuous production of corn, cotton, soybean, and winter wheat.^31,32^ An examination of crop species and temporal diversity found the average temporal crop species diversity, measured as the number of species within a rotation, in the US to be 2.1, with 60% of cropland planted to two or fewer crops.^32^

The most simplified US crop rotations are typically practiced on the most productive soils and in areas where optimal rainfall is achieved. Conversely, farmers with marginal land or suboptimal rainfall levels often employ more diverse rotational practices, likely out of necessity to maintain soil health and profitable production levels.^33^ Although the simplified nature of crop rotations in the heart of the US agricultural regions seems counterintuitive, it illustrates a “reactive” rather than “proactive” approach to rotational expansion. In response to economic and policy incentives, farmers choose to plant the crops with the highest values as often as possible until they are not able to maintain the desired production levels. Farmers may even see the value in diversifying crop rotations, but it may not be profitable for them to do so.^10^

## Conclusion

As shown in these results, HR weed populations have been increasing across the Prairies, but the rate at which new HR mechanisms are being reported is showing a slight decrease. The introduction of GMHT crops does not appear to have impacted the development of new HR weed populations and may in fact have helped to combat the development by replacing the use of other herbicides more likely to cause resistance issues. The rate of reporting of new HR weeds has decreased, with four of the past six years analyzed reporting zero new HR weeds. These results are reinforced by Kniss^11^ who found that the rate of new resistant weed species has remained the same or slightly decreased since the 1990s and came to a similar conclusion that GMHT crops, in general, had little to no impact on the development of HR weed populations. It is important to note, however, that this analysis only considers the first occurrence of a resistance mechanism and does not take into account the proliferation of these populations after discovery.

The increasing frequency and distribution of HR weeds previously reported, especially wild oats and kochia, continue to pose concerns as resistant biotypes are discovered in more Prairie fields.^15,17^ Left uncontrolled, these resistant populations pose substantial risk to crop production levels, and continued diversification and expansion of weed management practices is vital to managing this issue. However, diversification of crop rotations on the Prairies may have positively contributed to the relatively slow development of new HR weed populations over the past 30 years. Farmers have expanded the crop types they plant within their rotations to include pulses, oilseeds, and cereal crops. Within these crop types, many farmers are rotating varieties and HT traits to further aid in herbicide rotation. These management changes help to alleviate selection pressure for HR weeds, keeping the problematic proliferation of these populations at bay.

In comparison, the issue of HR weed populations has become more prevalent in the US over the past 30 years, where the majority of crop rotations are quite simple, especially among the most produced crop types. Although there are many factors to consider when comparing agricultural production in the two countries, including differences in climate, weed species, crop types, and agronomic practices, differences in rotational management may contribute to the different experiences with HR weeds.

Once resistant weeds have been selected, reversal of these genetic shifts within a population is not possible. However, with diverse, sustainable, and effective weed control measures, Prairie farmers have helped to keep HR weed populations at a manageable level. This is not to say that the expansion of resistant weed populations will not become a more pressing and urgent problem in the coming years. Farmers must continue to practice integrated weed management systems utilizing a variety of weed control methods to minimize the development of new resistance mechanisms. Continued investment into research and breeding programs to develop new crop varieties, herbicide options, and other weed management strategies will be necessary to maintain the current level of HR weed populations in the long term.^34^

## Notes

[a] While there is mutagenesis developed HT canola, GM varieties account for over 90% of production acres, which is why the article refers to HT canola as GMHT canola in most instances.

[b] Glyphosate and glufosinate canola varieties are GM, while imidazolinone tolerant varieties are non-GM.

[c] Bayer CropScience has commercialized canola varieties containing a combination of LibertyLink (glufosinate tolerant) and Roundup Ready (glyphosate tolerant) traits.
